# Paving the way for new and challenging matrix reference materials—particle suspensions at the core of material processing providing RMs for method development and method validation

**DOI:** 10.1007/s00216-023-05046-2

**Published:** 2023-11-21

**Authors:** Håkan Emteborg, John Seghers, Silvia García-Ruiz, Saioa Elordui-Zapatarietxe, Andreas Breidbach, Kamel Labibes, Jean Charoud-Got, Robert Koeber

**Affiliations:** 1 European Commission, Joint Research Centre (JRC), Geel, Belgium; 2 Diputación Foral de Bizkaia, Bilbao, Spain; 3 Ayam Sailing Europe, ASE, La Louvière, Belgium

**Keywords:** Reference material, Certified reference material, Homogeneity, Particles, Suspensions, Heterogeneity, Method validation, Repeatability, Spiking, Microplastics

## Abstract

Sufficient homogeneity of the certified parameter(s) over the whole fill series of a matrix reference material (RM) is a fundamental quality criterion. In practice, the heterogeneity of the target parameter is evaluated, whereby a relative value can be calculated of how much the target parameter is varying over the RM-batch. A high degree of homogeneity (low heterogeneity) is an inherent quality mark of a good RM. Here, we report how challenging matrix RMs were produced by using particle suspensions at the core of the material processing step. The examples of matrix RMs produced span from whole water reference materials for persistent organic pollutants, PM_2.5_-like atmospheric dust certified for specific ions to microplastic RMs. Most of these RMs were subsequently used in different phases of analytical method development or for method validation. Common to all these matrices is that they cannot be easily mixed, handled, or dosed to prepare larger sample batches. In all cases, a continuously stirred suspension of particles was used during material processing. In general, relative between-bottle heterogeneities from 1.6 to 6% were achieved for the target parameters in these matrix presentations. Concerning developments of new CRMs in emerging fields, the co-dependence between the availability of validated analytical methods with good repeatability and testing materials with a known and high homogeneity of the target parameter(s) becomes particularly challenging. This situation is an RM/Method causality dilemma. To overcome that hurdle, strategies are proposed for stepwise processes where RM producers and a network of analytical method developers could work hand in hand. In addition, development of a portfolio of inexpensive and well-homogenised common samples coupled with a reporting interface is suggested. This would benefit method developers and RM producers alike. As more and more data is compiled for a specific matrix, it paves the way for new and challenging RMs that can later be used by a wider community.

## Introduction

Reference materials (RMs) and especially certified reference materials (CRMs) are efficient tools in the analytical laboratory. They can be used for validation of analytical methods, to prove trueness, and provide metrological traceability of the measurement results. They can also be used for training of new laboratory staff to check their performance or for the preparation of control charts. The terms RM and CRM are defined in ISO Guide 30 [1]. Consequently, a reference material is a *material*, sufficiently homogeneous and stable with respect to one or more specified properties, which has been established to be fit for its intended use in a measurement process. A certified reference material is a *reference material* characterised by a metrologically valid procedure for one or more specified properties, accompanied by an RM certificate that provides the value of the specified property, its associated uncertainty, and a statement of metrological traceability [1]. One of the key statements is, “sufficiently homogeneous” which means that the reference material itself should not contribute excessively to the variability of the measurement results. If a reference material is inhomogeneous, there can only be limited meaningful comparison between different analytical results for a target parameter of interest. Hence, a high degree of homogeneity of the target parameter in a reference material is essential.

**Fig. 1:**
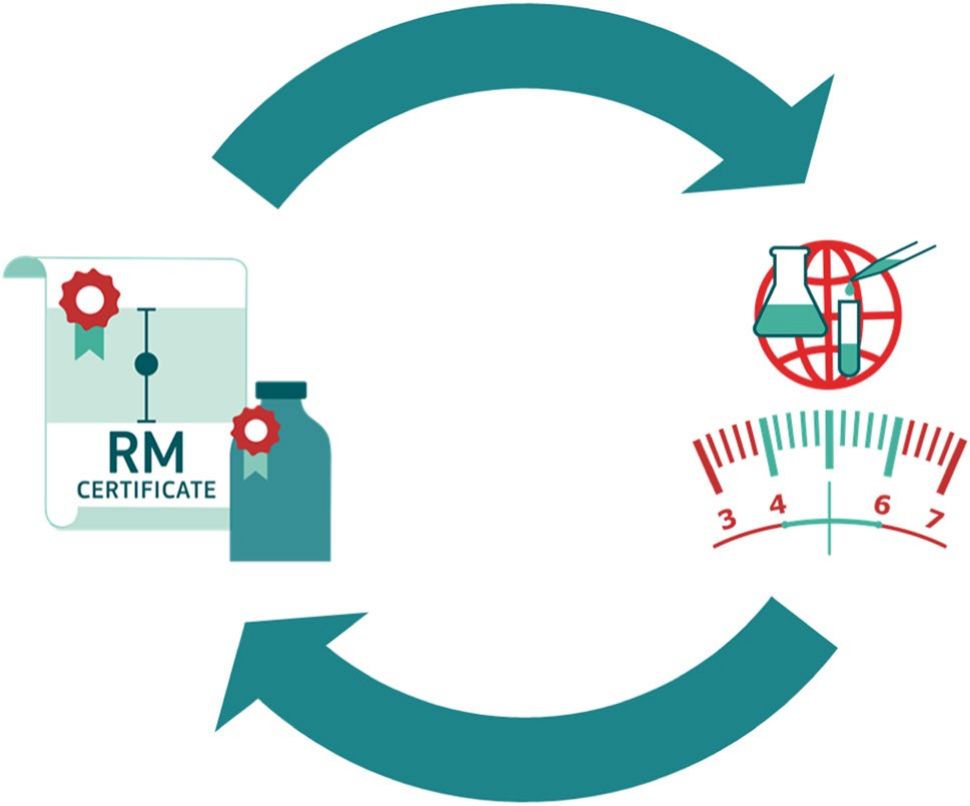
The RM/Method causality dilemma. Accurate and validated analytical methods are required for production of RMs while RMs are needed for validation of analytical methods. There is an apparent circular argument: what comes first? The RM or the method, the chicken or the egg?

A particularly challenging situation occurs at the frontline of new areas of measurement, where new and developing analytical methods are often not fully validated. The inter-dependence between availability of RMs and validation of new methods needed for testing of such reference materials during their production is often overlooked. Evolving measurement communities understandably request (certified) reference materials probably without realising that production of such RMs is largely dependent on access to reliable and validated analytical methods. It is a circular argument where you in principle cannot have one without the other, it’s like the chicken or the egg; what came first? We can call this the RM/Method causality dilemma as shown in Fig. 1. It should be noted that the validity of the RM/Method causality dilemma is mainly associated with RM preparations which rely on interlaboratory comparisons for value assignment of the certified parameters.

Likewise, in proficiency testing (PT) or interlaboratory comparisons (ILC), access to sufficiently homogeneous test items is also mandatory. This article will describe possible strategies for an RM producer to overcome such hurdles in an incremental fashion and highlight this inter-dependence for analytical method developers. Additionally, in a recent work on a microplastic (MP), RM demonstrates how improved analytical methods reveal better homogeneity of a reference material than initially established [2, 3]. Under all circumstances, access to homogeneous testing materials is vital in the early steps of such an incremental development process.

Occasionally, RM producers are confronted with requests for reference materials that are difficult to process. It can also be that matrices and starting materials may be hard to source in sufficient quantities for different reasons. Given that enough starting material is available, the way forward is normally to homogenise the bulk material before filling and thereafter accurately fill the material in larger series. Clearly, painstaking manual work to fill up to 50 or perhaps 100 sample units is possible but not feasible for larger amounts of samples [4]. Over the years, the European Commissions’ Joint Research Centre in Geel, Belgium, has prepared several new types of reference materials whereof one is a certified reference material for PM_2.5_-like atmospheric dust, which was described in two separate papers [5, 6]. The other two examples discussed in this work encompass whole water reference materials for organic pollutants in environmental water samples [7–13] and two RMs for mass and/or particle number of microplastic in water [2, 3, 14]. These examples cover both the difficulty of access to sufficient amounts of a suitable starting material and inherent challenges associated with processing. These materials were subsequently assessed using analytical methods under development or used for methods subjected to validation.

## Strategies for processing of new and challenging matrix RMs

### Whole water reference materials for EU priority substances

The first attempts to use slurries and suspensions of particulate matter to process reference materials at JRC-Geel were undertaken for a suite of whole water reference materials during the ENV08 project (traceable measurements for monitoring critical pollutants under the European Water Framework Directive funded by the European Metrology Research Programme, EMRP [10–13]). According to the (WFD) Directive 2000/60/EC, Directive 2008/105/EC, and amending Directive 2013/39/EC, priority hazardous substances (PS) must be monitored in water and biota by the Member States to ensure that the environmental quality standards (EQS) are met [7–9]. Therefore, RMs were prepared for the analysis of polybrominated diphenyl ethers (PBDEs), polycyclic aromatic hydrocarbons (PAHs), and tributyltin (TBT) in environmental whole water samples [10–13]. These materials and analytical methods developed in parallel were addressing the need for reference materials and validated methods to underpin the European Water Framework Directive and its amendments [7–9]. The main challenge with that project was the preparation of batches of homogeneous whole water reference materials where the priority substances were present at ultra-trace levels relevant for environmental monitoring [8]. In whole water (non-filtered water), the PS are bound to natural ligands, e.g. suspended particulate matter (SPM) and humic acids, just like in authentic environmental water samples which is evidently why the legislation is formulated in that way. The concept of preparing such whole water test materials was published in 2015 by Elordui-Zapatarietxe et al. [10]. Additional papers addressed a sample container study testing different bottles for containment, results of interlaboratory comparisons, and estimation of uncertainty budgets for the target parameters of PS in the whole water test materials [11–13]. In addition, material supply to CEN technical committee M424/TC230 for the development and validation of methods for three analytical standards was also undertaken using the developed approach [15–17]. This material supply is a good example how a sufficiently homogeneous reference material can help a measurement community to validate methods. Those methods are now disseminated in standards EN 16691:2015 (PAH) and EN 16694:2015 (PBDE) and CEN/TS 16692:2015 (TBT) (TS stands for a technical specification) [15–17]. These standard methods can evidently be used for the characterisation of candidate reference materials.

### PM_2.5_-like airborne dust CRM, ERM-CZ110 for specific ions

In the subsequent project, an airborne PM_2.5_-like dust CRM, (ERM-CZ110) was produced to support the implementation of existing EU legislation Directive 2008/50/EC, on ambient air quality and cleaner air for Europe [18, 19]. The production was underpinned by measurements using methods based on extraction with water, ion chromatography with conductivity detection, and inductively coupled plasma atomic emission spectrometry that had already been developed by CEN on air-sampled PM_2.5_ [20, 21]. In this case, the analytical methodology and sampling procedures were not a limiting factor and the main challenge was access to sufficient amounts of a starting material. It would take close to 1000 years to collect 300 g of PM_2.5_ from the air using one sampler operated under standardised conditions [21]. Therefore, another solution had to be found.

A previous approach of jet-milling a tunnel dust to the required particle size was not possible as employed for PM_10_-like materials (ERM-CZ100 and ERM-CZ120) developed some 10 years earlier [22, 23]. Two other routes were then investigated, where a suitable method based on a shock frozen suspension of PM_2.5_-like particles spiked with the ions of interest was devised [5]. To obtain the PM_2.5_-like particle suspension, jet-milled tunnel dust of PM_10_-like particles had settled for 72 h so that larger particles were at the bottom of the vessel. The particles remaining in suspension were thus sufficiently small to fulfil the definition of PM_2.5_-like particles [21]. Afterwards the suspension was continuously stirred as it was pumped out using a peristaltic pump and was dropped into liquid nitrogen to produce ice kernels of 5- to 8-mm diameter which were then freeze-dried. After freeze-drying, the resulting powder was transferred directly to a glovebox filled with inert and dry N_2_ for subdivision and filling in vials since the PM_2.5_-like material could not be handled in the open air due to excessive water uptake. In total, four freeze-drying cycles of about 212 kg of ice kernels from the same suspension were accumulated resulting in 400 g of PM_2.5_-like dust. The homogeneity of the suspension was proved as the content of ions in the dust from four freeze-drying cycles was uniform. Although this PM_2.5_-like material was not authentic air sampled dust, it behaved in the same way as air-sampled PM_2.5_ during extraction [6].

### Reference materials for microplastics in water samples

There are currently no certified matrix reference materials available for purchase that are certified for mass and/or number concentration of microplastic particles. These environmental pollutants are of increasing and significant concern and there is evidence for presence of MP in different sample types from humans [24–26].

The measurement communities of microplastics are requesting (C)RMs since many years but it has also been shown in two inter-laboratory comparisons conducted by WEPAL Quasimeme and the JRC/BAM (Bundesanstalt für Materialforschung und -prüfung, DE) that harmonisation of analytical methods for the analysis of microplastics is necessary [4, 14]. As with all such emerging measurement challenges, access to sufficiently homogeneous test materials to underpin such harmonisation and method validation efforts is crucial. During the last years, work has been undertaken at the JRC in order to pave the way for the production of such RMs [2, 3]. To date, the two JRC publications cover the methodology and further characterisation of the polyethylene terephthalate PET in drinking water RM used in the JRC/BAM inter-laboratory comparison [14].

As far as the authors know, there is no simple or automatic way of weighing and accurately dosing dry PET particles (> 20 to 300 µm Feret_min_) in small amounts (200–400 µg) with ± 3–5% variability into suitable containers. This is mainly because plastic particles are highly electrostatic. Based on the experiences from the whole water and dust projects, it was evident that handling of microplastic particles would be easier if in suspension also allowing upscaling. The addition of a surfactant (Triton X-100) to the 10–25% NaCl (m/m) suspension containing MP under constant mixing in a beaker helps to overcome the hydrophobicity and the MP particles remain in solution. After freeze-drying, ultra-microbalances were used to assess the homogeneity of the RM after filtration and weighing the dissolved salt pellet containing the PET particles, which were captured on silicon membrane filters [2]. By using a balance for characterisation of the amount of microplastics, metrological traceability to the SI system was achieved as well as trueness check of the actual content in the samples. Subsequent efforts using quantitative ^1^H-NMR that had shown promising features in the JRC/BAM inter-laboratory comparison provided more information about the homogeneity of the RM used [3, 14]. Results obtained by ^1^H-NMR are also traceable to the SI system with the additional advantage of being specific for PET [3].

Other RMs for microplastics have specifically been prepared and used to implement the Erasmus Maris concept. The Erasmus Maris concept is a citizen science project that aims to engage upper-secondary schools in collaborative scientific research related to marine conservation. Quality assurance and training of students and their teachers on the analytical method is achieved with a dedicated reference material. Subsequently, the measurements of microplastics in waterbodies nearby schools participating to the Erasmus Maris’ activities have the necessary QA underpinning [27].

These RMs were prepared and adapted for water samples coming from surface sampling of ocean and inland waters as the main polymers like polyethylene (PE) and polypropylene (PP) float on the water surface (density < 1 g/cm^3^). Sampling is performed using Manta nets (given that name because of their shape, e.g. plankton nets with a mesh size of 300 µm) which are dragged or kept in the flow of a river to cover a specific surface area/volume. The microplastic particles are captured at the end of this Manta net. In this RM, the microplastic particles were > 300 µm to 1 mm and made up of PE and PP. A 10% (m/m) NaCl 0.1% surfactant suspension containing MP was prepared from which sub-portions were pipetted. Mixing was done in a 3-L glass beaker using an overhead stirrer/stainless steel propeller. An excess volume of suspension was produced to allow uniform mixing conditions until the last vial was filled. The pipet was inserted against the flow of the vortex during aliquoting from the suspension. It is also important to use a pipet tip with an opening that is large enough not to discriminate the larger MP particles. In this way, it was possible to prepare > 500 units of an RM that was sufficiently homogeneous for further use.

A user-friendly and validated sample preparation and manual counting methodology was also developed to allow secondary schools to analyse sea and river water samples in their own chemistry laboratories. The resulting RM described above, incorporating a specific number of larger PE and PP microplastic particles, was used for spiking of natural filtered waters which had a substantial load of organic material. Recoveries were found to be relatively high and consistent after sample treatment with sodium hypochlorite to digest the organic components of biological origin. These spiking experiments were part of the method validation engaging two different operators on two different days for two different matrices of filtered sea and river water (*n* = 24). The recoveries were 86 ± 11% (*n* = 12) in seawater and 84 ± 12% in river water (*n* = 12). These results provide a good prospect that also incipient MP particles from non-filtered water samples can be properly quantified.

### Achieving sufficient homogeneity and its assessment

Table 1 shows the relative between-unit heterogeneities (u_bb_) of the RMs described in this work. To determine those u_bb_ values, methods of analysis are necessary which are sufficiently precise for the required test portion intake (minimum sample intake). For the materials described herein, two different situations apply: (1) the material unit has sufficient internal homogeneity, which allows sub-sampling and repetition, or (2) the contents of the material unit are too heterogeneous for sub-sampling and must be analysed in “one-shot”.

**Table 1:** Homogeneity expressed as relative between-bottle heterogeneities for target parameters in the different reference materials discussed in this work

Reference material	Target parameter and unit	Relative between-bottle heterogeneity u_bb_ (%)	References
ERM-CZ110	Major ions, g/kg^y^		[5, 19]
	Na^+^	2.1	
	K^+^	3.8	
	Ca^2+^	3.6	
	Mg^2+^	6.0	
	Cl^−^	2.7	
	NO_3_^−^	3.1	
	SO_4_^2−^	4.5	
Whole water RM used in CEN / ENV08 studies^a^	PBDEs, ng/L^n^		[11]
	BDE28	8.7	
	BDE47	7.8	
	BDE99	3.7	
	BDE100	3.5	
	BDE153	4.7	
	BDE154	4.6	
Whole water RM used in CEN / ENV08 studies^b^	PAH, ng/L^n^		[11]
	N	4.1	
	A	2.2	
	F	2.6	
	B(b)F	2.0	
	B(k)F	4.8	
	B(a)P	2.2	
	I	1.6	
	B(ghi)P	2.4	
Whole water RM used in CEN / ENV08 studies^c^	TBT, ng/L^n^	3.1	[11]
Microplastics RM for PET in water^d^	Mass of PET, μg^n^	14	[2]
Microplastics RM for PET in water^d^	Mass of PET, μg^n^	7.9	[3]
Microplastics RM for PE and PP in water^e^	Number of PE and PP particles^e,n^	5.0	This work

^a^Jet-milled sediment from Speibeek, BE, ^b^jet-milled industrial soil from BAM, ^c^jet-milled sediment BCR-646 from JRC, ^d^20–300 μm (Feret_min_), ^e^300 μm to 500 µm (Feret_min_), ^y^yes, sub-sampling possible, ^n^no, sub-sampling *not* possible

ERM-CZ110 [19] is a material for which situation (1) applies. Because of the low particle size and the extensive mixing and processing, test portion sizes as small as 5 mg were representative enough for the whole test unit of 150 mg. This allows for a homogeneity study design with replicate measurements that can be evaluated using analysis of variance (ANOVA) as described in ISO Guide 35 [28]. The repetition of measurements per unit allows determination of the repeatability (within-unit variance) of the method of analysis as well.

Situation (2) applies to all the described water reference materials. Here a very small amount of solid material, being finely milled sediment or microplastic, is surrounded by a much larger amount of water. The added solid material does not go into solution or does not form a uniform suspension. Therefore, it is practically impossible to obtain a representative sub-sample. No repetition is possible and u_bb_ cannot be determined with ANOVA. Here the repeatability standard deviation of the method of analysis needs to be known with sufficient confidence from independent validation studies in order to establish the between-bottle heterogeneity. The method repeatability must thus be established in separate experiments.

The value that can be determined for u_bb_ is not only dependent on the “true” heterogeneity between RM units but also on the precision of the method analysis employed for the testing. Even in the very hypothetical case of no heterogeneity between units, u_bb_ would therefore not be zero. For RMs for which situation (1) applies, a somewhat lower precision of the analytical method can be compensated for by performing a higher number of replicate measurements of the same sample unit. Lower precision usually means less operational effort but this approach is not really an option for analysis methods to test RMs for which situation (2) applies. In those cases, a method with lower precision will directly lead to higher apparent heterogeneities between units and doubt about the actual quality of the material.

An example of that effect can be seen in the microplastics reference material for PET in drinking water of which more than 500 samples were prepared. “One-shot” analysis had to be applied. Initial evaluation using ultra-micro balances of fourteen samples resulted in a relative between-bottle heterogeneity of 14% [2]. Subsequent work using quantitative ^1^H-NMR revealed a relative between-bottle heterogeneity of 7.9% (n = 10) for the same reference material [3]. The second relative heterogeneity result is about half of the first for the same reference material. This shows that an improved precision of the method of analysis provides a sharper detail about the actual heterogeneity. For the microplastic RM containing the larger PE and PP particles, a separate method validation study provided a method repeatability of 5.6% when counting ten samples containing 50–80 particles manually by two different operators using the devised methodology. In that case, the square of the method repeatability was subtracted from the square of a relative standard deviation of 7.5% that was obtained by measurement of eight samples spread out over the whole RM batch. Consequently, taking the square root following subtraction, a value of 5.0% for the between-bottle heterogeneity was obtained. This value is reported in Table 1 as an estimate of the u_bb_ for that material preparation.

Relative heterogeneities of 1.5 to 4% are also common for many parameters in certified reference materials of powder RMs, which are much less demanding to process, e.g., for matrices where dry-mixing of free-flowing powders is possible. Consulting certification reports for pesticides in soya (ERM-BC700), vitamins in milk powder (ERM-BD600), and trace elements in kidney (ERM-BB186) show such heterogeneities for a variety of organic compounds and elements [29–31]. The results for between-bottle heterogeneity reported in Table 1 are good evidence of how efficient slurries and suspensions are for homogenisation of target parameters in challenging matrices as they are comparable with between-bottle heterogeneities for parameters in powder matrices. The improved analytical method repeatability of ^1^H-NMR compared to the direct use of ultra-micro balances revealed that the RM used in the JRC/BAM intercomparison was more homogeneous than previously established [2, 3, 14]. Hence, the incremental improvement of analytical methods for testing of material preparations was also demonstrated and it shows a way out of the RM/Method causality dilemma.

## Step-by-step approach to resolve the RM/Method causality dilemma

### Spiking

The use of fortified test samples to determine recovery rates is an important means in all analytical method development as shown in Table 2. Normally, this is done by spiking a well-characterised target analyte with known concentration, purity, and identity in solution to a blank matrix aiming to analyse authentic matrix samples with the same but *naturally embedded* analyte in subsequent steps of method development. Well-described spiking protocols should be employed including specific equilibration times and high and low spike levels when adding the spike solution to a blank matrix. High spike recoveries are an indication that it would be possible to achieve high extraction efficiencies of embedded analyte although this is not guaranteed. For that reason, it is mandatory that the spikes behave like the embedded analyte when interacting with the matrix before and during sample preparation. This may be hard to verify but exhaustive extraction protocols based on repetition of several extraction cycles can reveal how close one is to a quantitative extraction efficiency of a naturally embedded analyte.

**Table 2:** Incremental development steps between an RM producer and a network of collaborating laboratories leading to a reference material development in a naturally incurred matrix. The approach is suitable for emerging fields of measurements where there is a lack of harmonised and validated methods and reference materials

	RM producer (Provider of different types of samples and materials in an incremental fashion)	Network of collaborating laboratories (Providers of analytical results using promising methods and techniques to measure target parameters in the samples) -
Step	Type of sample	What can be evaluated?
1	Control sample (QC) stock solution for dilution (e.g., common calibrant of known purity gravimetrically prepared). Blank solvent	Linear range, LOD/LOQ, blanks sensitivity/selectivity
2	Control sample gravimetrically prepared of known purity	Bias upon calibration (laboratories required to use their own calibration standards of different origin) Purity of calibrants
3	Blank matrix and spiking protocol (high / low / time for equilibration) using a well-characterised spiking solution	Recoveries, accuracy and trueness (to some extent) in the development phase
4	PT-material, (non-spiked incurred matrix with incipient analyte, preferably with reference value tested for homogeneity and stability	Z-scores, zeta-scores, satisfactory results / unsatisfactory results extraction efficiencies Screening for laboratories to be used in step 5
5	Candidate CRM, incurred matrix containing target analyte at relevant level(s)	Homogeneity, stability characterisation (6–8 acceptable data sets for characterisation, value assignment with uncertainty)
	RM producer with new CRM available for sale	External customer laboratories
6	CRM & certificate	Validation of methods ISO/IEC-17025, control charts, trueness check, metrological traceability, measurement uncertainties

Even the spiking step itself might nevertheless be quite challenging, as was the case for the first preparation of a large number of uniform microplastic samples that were later used for spiking of drinking, sea and river waters [2]. For the PM_2.5_-like dust and for the whole water samples for EU priority substances, the spiking process was not a direct spike added to a blank matrix either. Therefore, the spiking processes employed in this work should be described in more detail.

In the case of the PM_2.5_-like dust, spiking of specific ions in solution took place directly into the particle suspension whereby they adhered to the particle surfaces [5, 19]. The final matrix presentation itself was a dry particulate matter where the spiked ions were adsorbed on top of lower background levels of the same ions already associated with the particles. For the whole water samples, contaminated soils and sediments were jet-milled down to top particle sizes around 10 μm, which is similar to top particle sizes for SPM in natural waters. In this case, no direct spiking of the target parameters took place. The content of specific priority substances (PS) in the whole water sample was calculated by multiplying the mass of SPM reproducibly added to each water sample (e.g., from 20 to 200 mg) with the content of PS per gram of dry SPM [10–13]. The priority substances already present on the SPM were tenaciously bound to the SPM as shown in leaching experiments [10]. This made accurate dosing of PS using slurries possible. If a large fraction of the PS would have been liberated into the water phase in the initial slurry step, this approach would not have worked. Spiking was consequently done with a specific mass of SPM from 20 to 200 mg/l (e.g., per sample container of 1 L) and since the PS were literally immobilised on the solid carrier, it was effectively a direct spike of PS to the water sample. In case of microplastics, the preparation of a large number of samples with sufficient homogeneity where the MP particles of PET, PE, and PP were immobilised in a salt cake, provided the actual spikes that were added to drinking water, filtered natural sea, and freshwater samples as already described above [2, 3]. For the analysis of MP in sea and freshwater samples collected using Manta nets, a digestion step using sodium hypochlorite was necessary. A recovery of PP and PE MP particles obtained during method validation was globally 85 ± 11% (*n* = 24) in sea and river water samples. Likewise, the MP salt cakes with PET were directly transferred (spiking) to 1 L of pure water using a surfactant to mimic MP in drinking water sample [2, 3, 14]. In this case, no digestion using sodium hypochlorite was necessary since the matrix was pure water.

Figure 1 shows the RM/Method causality dilemma where validated methods are necessary for production of certified reference materials and certified reference materials are used for validating methods. Table 2 lists suggestions for steps that can be taken in an incremental manner between an RM producer and a network of laboratories in early stages of an emerging measurement challenge. Skilled analytical chemists certainly go through such method development steps routinely but the certification of an RM requires a *network of competent laboratories* employing validated and harmonised methods. Table 2 intends to show how an RM producer can develop and engage such a network. Starting simple and subsequently adding increased complexity is the main feature although it may be necessary to repeat steps if problems occur. Each step should provide enough knowledge and information that can then be applied to design subsequent steps. The steps shown in Table 2 help to resolve the RM/Method causality dilemma shown in Fig. 1 so that a CRM eventually can be produced as shown in the last step in Table 2.

## Outlook

This Trends article is describing the interface between reference material processing developments, analytical method developments, and the necessary interlinked incremental steps for subsequent reference material production. Three examples from different areas of measurement (organic trace analysis, microplastics, and air particulate monitoring) show similarities and differences of the approaches. Common to all material developments is that they were challenging matrices to source, process, and handle. In addition, central for all these material preparations, is a particle suspension that was continuously mixed or agitated from which sub-portions were taken by pipetting or pumped out of the suspension during processing for aliquoting. The work described is also an example of how approaches in one area can be transferred to another field of analysis, e.g., from whole water RMs containing organic pollutants bound to natural ligands via PM_2.5_-like atmospheric dust to microplastic RMs [2, 3, 5, 6, 10–13]. In addition, these materials were in case of the microplastics and whole water materials, helping measurement communities to improve and/or validate analytical methods.

Access to homogeneous test materials is necessary for all laboratory networks that wish to harmonise their methods or compare their results and evidently also for Proficiency Testing (PT) exercises. It was suggested by Wise that “common samples” could help laboratories to more rapidly achieve comparability between their measurement results [32]. We suggest that a reporting interface for analytical results should be coupled with such a pool of common samples where anonymised measurement results would be publicly available for all. This would make it possible for laboratories to select a matrix of interest where data already exists as soon as the results database is populated. The RM producer would also benefit directly as more and more data accumulates. The producer can learn if measurement results have good comparability for methods and analytes that could be of interest for future CRM developments. For the material supplier, it must therefore be possible to identify the laboratories associated with specific data sets for potential collaboration and future contracting of a network of laboratories. The combined efforts between laboratories and RM provider(s) should shorten the time to market for new CRMs once such a system is consolidated.

Such common samples or common materials also help to support another much wider development that has taken place in analytical chemistry over the last two–three decades as described by Adams and Adriaens [33]. In that article, it is clearly outlined how analytical chemistry nowadays has become an analytical science that has the capability to generate an enormous amount of data per sample and that the traditional metrological-driven approach to “perform analytical chemistry” is only partially applicable as shown in Fig. 3 in that work. Hence, the analytical communities would most likely welcome availability of inexpensive homogeneous common samples for non-targeted analysis (NTA) as well [34]. As a testimony to that general development in analytical chemistry, the impressive capability of the so-called QuEChERSER method described by Lehotay shows that sample preparation for several hundreds of compounds can be done and analysed in one run, e.g., pesticides, mycotoxins, environmental contaminants, and veterinary drugs [35].

Currently, no RM producer (or PT provider for that matter) offers such common and homogenised samples of different matrices coupled with a reporting database to be used for the purposes outlined here. As was discussed above concerning spiking (step 3 in Table 2), even a common blank matrix would be useful as these can be used for spike recoveries. The advantage would be that laboratories still work on common samples regardless if they are naturally incurred or not. Many publicly funded RM producers already have the necessary building blocks available for such a development, e.g., reporting interfaces and databases for measurement data and material processing facilities [36]. Generally, the RM producer can utilise common processing techniques to mill, thoroughly mix, and fill the matrices in suitable containers without a need for extensive additional measurements. Although, long-term storage at − 20 °C of these materials would be advisable including a cooled shipment.

A similar concept exists for environmental specimen banking [37]. In some cases, biological materials of environmental origin are cryogenically milled and stored over liquid nitrogen with a continuous addition of the same category of samples for monitoring of long-term trends in the environment. Hence, the purpose is different and the storage option chosen is more expensive but it adheres to a similar concept of measurements of *the same kind* of “common samples”. Yet another approach was described by Roebben et al. where the term representative test material (RTM) was chosen for a category of common samples. That work was mainly focussed on developments of methods for measuring properties of nanomaterials but the term RTM could also be extended to the common materials described here [38].

Finally, the demand for matrix reference materials and/or test materials is much higher than can be met by RM producers. The route to widen access to common samples as suggested by Wise is certainly worth to explore further [32].

Evidently, disclaimers about the quality of these common samples must be provided so that it remains clear that values reported into the database are not certified. The information available per material is also limited at the beginning but given time, highly useful information for both RM producers and measurement communities will be generated. A good starting point for selection of common samples in the food area would be the compositional AOAC food triangle [39, 40]. Food matrices could therefore make up the first sets of common samples as the composition of any matrix is directly linked to analytical method performance.

Perhaps the time has come to diversify the materials available in the RM-catalogues for the benefit of laboratories and producers alike?
